# Target-conditioned flow-matching policy for citrus harvesting robot pre-grasp approach behavior learning

**DOI:** 10.3389/fpls.2026.1853570

**Published:** 2026-06-16

**Authors:** Luo Lei, Han Li, Zhijiang Zuo

**Affiliations:** School of Intelligent Manufacturing, Jianghan University, Wuhan, China

**Keywords:** citrus harvesting robot, behavior learning, imitation learning, target-conditioned policy, flow matching, multi-step trajectory prediction

## Abstract

**Introduction:**

Fruit harvesting in natural orchards remains challenging because target fruits are distributed in cluttered and unstructured environments. In the pre-grasp approach stage of multi-fruit citrus harvesting, three issues are particularly critical: limited demonstration data, target ambiguity, and the need for stable and precise local approach motions.

**Methods:**

To address these issues, this study proposes a Target-Conditioned Flow-Matching Policy (TCFM Policy), which integrates image observations, robot state history, and explicit target geometric conditions, uses a dual-branch visual representation to encode both global scene context and local end-effector details, and predicts future multi-step TCP trajectories through conditional flow matching. To reduce overfitting to global appearance under small-sample conditions, a target-oriented visual augmentation strategy is further introduced for the global branch during training.

**Results:**

A real-world dataset containing 160 valid demonstration episodes was collected on a UR5-based citrus harvesting platform using VR teleoperation. In 50 target-specified multi-fruit trials, the full model achieved a success rate of 76%, a target-picking error rate of 4%, and a picking-point offset rate of 20%.

**Discussion:**

A fairness-aligned comparison with a target-conditioned diffusion-policy baseline further shows that the proposed method achieves lower offline trajectory error and better online target-specified approach performance under the same training setting. Ablation results indicate that the ROI branch mainly improves final alignment, while the target-oriented augmentation mainly improves target consistency. These results indicate that explicit target conditioning, dual-branch visual encoding, and conditional flow matching jointly support accurate target selection and relatively stable pre-grasp approach execution in small-sample multi-fruit citrus scenes.

## 1 Introduction

Fruit harvesting is one of the more challenging tasks in agricultural robotics (Li et al., 2022; Xiao et al., 2023). Unlike structured industrial operations, fruit-tree crops such as citrus typically grow in open and unstructured environments, where harvesting is affected by branch and leaf occlusion, natural illumination changes, complex background textures, fruit-scale variation, and uncertain stem pose (Yang et al., 2023). In recent years, advances in deep visual perception, agricultural manipulators, and end-effectors have promoted the development of fruit-harvesting robots. However, current systems are still largely limited to specific operating conditions, and their robustness and generalization in complex environments remain insufficient (Mail et al., 2023; Jin and Han, 2024).

Most existing harvesting robots follow a modular pipeline consisting of perception, target localization, path planning, and end-effector execution (Tan et al., 2025; Lin et al., 2026). At the perception level, researchers have continued to improve YOLO-based models to enhance fruit detection and localization under complex natural conditions. For example, Li et al. proposed an improved YOLOv8n-seg-based method for precise citrus fruit and stem segmentation, and further combined geometric constraints to localize stem picking points for robotic harvesting (Li et al., 2025). Yin et al. investigated key technologies of tomato-picking robots based on machine vision, integrating an improved YOLOv8n-based detection and spatial localization method with a non-contact cavity-type end-effector to support stable tomato harvesting (Yin et al., 2025). Ma et al. further introduced AHG-YOLO based on YOLOv11n for occluded fruit detection in complex pear-orchard scenes (Ma et al., 2025). In system coordination and task planning, Li et al. developed a multi-arm apple harvesting system that integrates precise perception, task allocation, and coordinated control (Li et al., 2023). Chen et al. investigated dynamic visual servoing to address the efficiency bottleneck of stop-and-go orchard operation and to support continuous motion and continuous harvesting (Chen et al., 2024). Hou et al. combined binocular vision and stem detection to localize citrus picking points (Hou et al., 2024). Wang et al. proposed a fast planning method based on pre-picking points and a quadtree to reduce manipulator path-search time. More recent citrus harvesting systems have further combined 6D pose estimation with grasping and cutting action generation, and have demonstrated end-to-end harvesting performance in real scenarios (Wang et al., 2022). These studies have advanced harvesting robots from fruit recognition toward practical field operation, while also showing that modular systems remain attractive from an engineering standpoint in constrained scenarios.

However, at the path-planning level, conventional modular and hard-coded solutions still rely heavily on hand-crafted intermediate representations and heuristic rules. Once crop variety, tree architecture, camera pose, or end-effector design changes, the system often requires redesign of picking-point determination logic and approach constraints. In addition, it is difficult to fully encode the nuanced operational skills accumulated by human workers for dealing with complex branch topology. A pipeline based only on detection boxes and geometric planners often fails to fully exploit fine-grained visual information that is directly relevant to action decisions, such as the spatial relationship between the target and the end-effector, local occlusion changes, and instantaneous reachability differences (Gao et al., 2024; Choi et al., 2025). With the development of temporal models, behavior cloning has provided an alternative route to harvesting policy learning beyond manual rule design.

The review by Mahmoudi et al. shows that behavior cloning has already been applied to agricultural navigation, path tracking, grasping, and harvesting tasks, mainly because it can learn control regularities directly from expert demonstrations and is therefore suitable for agricultural environments that are difficult to model precisely (Mahmoudi et al., 2024). LSTM-based behavior cloning is a representative early approach (Rahmatizadeh et al., 2018), where Rahmatizadeh et al. used long short-term memory networks to process action sequences. However, such methods have difficulty handling multimodality in complex tasks. Later studies introduced mixture density networks (Ho et al., 2020)or discretized continuous action spaces to capture multi-modal action distributions, but these approaches are often limited by distributional assumptions or by the loss of precision induced by discretization in high-dimensional continuous action spaces. With the rise of generative models, especially the success of denoising diffusion probabilistic models in image generation (Ho et al., 2020), robot control has gained a new set of tools. Chi et al. proposed Diffusion Policy (Chi et al., 2025), which formulates visuomotor policy learning as a conditional diffusion process and achieves strong performance in multi-task manipulation. Rouxel et al. and Chisari et al. further introduced flow matching and conditional flow matching into robot imitation learning (Rouxel et al., 2024), indicating that such generative approaches are better suited than conventional regression-based temporal models for representing multi-peak trajectory distributions and long action chunks.

Although behavior cloning offers a promising alternative to hand-engineered harvesting systems, existing methods cannot be directly transferred to agricultural harvesting. The main reason is that agricultural harvesting is jointly constrained by data scarcity, target ambiguity, and the need for precise local manipulation. On the one hand, data collection in agricultural harvesting is seasonal, expensive, and highly scene-dependent. Complete demonstrations often require mature fruits, suitable weather, an available test site, and a synchronized robot platform, making it difficult to construct large-scale and standardized datasets that cover diverse scene variations (Kim et al., 2025). On the other hand, in natural scenes with multiple targets, foliage occlusion, and complex backgrounds, end-to-end behavior cloning based only on images and robot states easily suffers from target ambiguity when explicit target constraints are absent, leading to compromise or averaged actions. Under small-sample conditions, this issue further causes the model to memorize demonstration trajectories or background appearance rather than focus on visual cues that are truly relevant to harvesting decisions. In addition, citrus harvesting requires high local perception accuracy and stable trajectories during the final approach to the target, which further limits the applicability of generic behavior-cloning methods in agricultural scenarios.

These constraints indicate that a harvesting policy should answer three questions simultaneously: which fruit should be harvested, which visual cues are most relevant for the decision, and how the future approach trajectory should be generated from limited demonstrations. To address these issues, this study proposes a Target-Conditioned Flow-Matching Policy (TCFM Policy). The main contributions are as follows.

1. A target-conditioned visuomotor policy is introduced to reduce target ambiguity in multi-fruit pre-grasp harvesting by incorporating explicit target geometric information into trajectory generation.
2. A dual-branch visual representation is constructed from global images and ROI images, and a target-oriented visual augmentation strategy is designed to improve visual robustness and target consistency under small-sample conditions.
3. Future TCP approach trajectory generation is formulated as a conditional flow-matching problem to improve multi-step trajectory consistency and task executability in precise approach operations.

## 2 Materials and methods

### 2.1 Target-conditioned behavior learning for citrus picking

**Figure 1:**
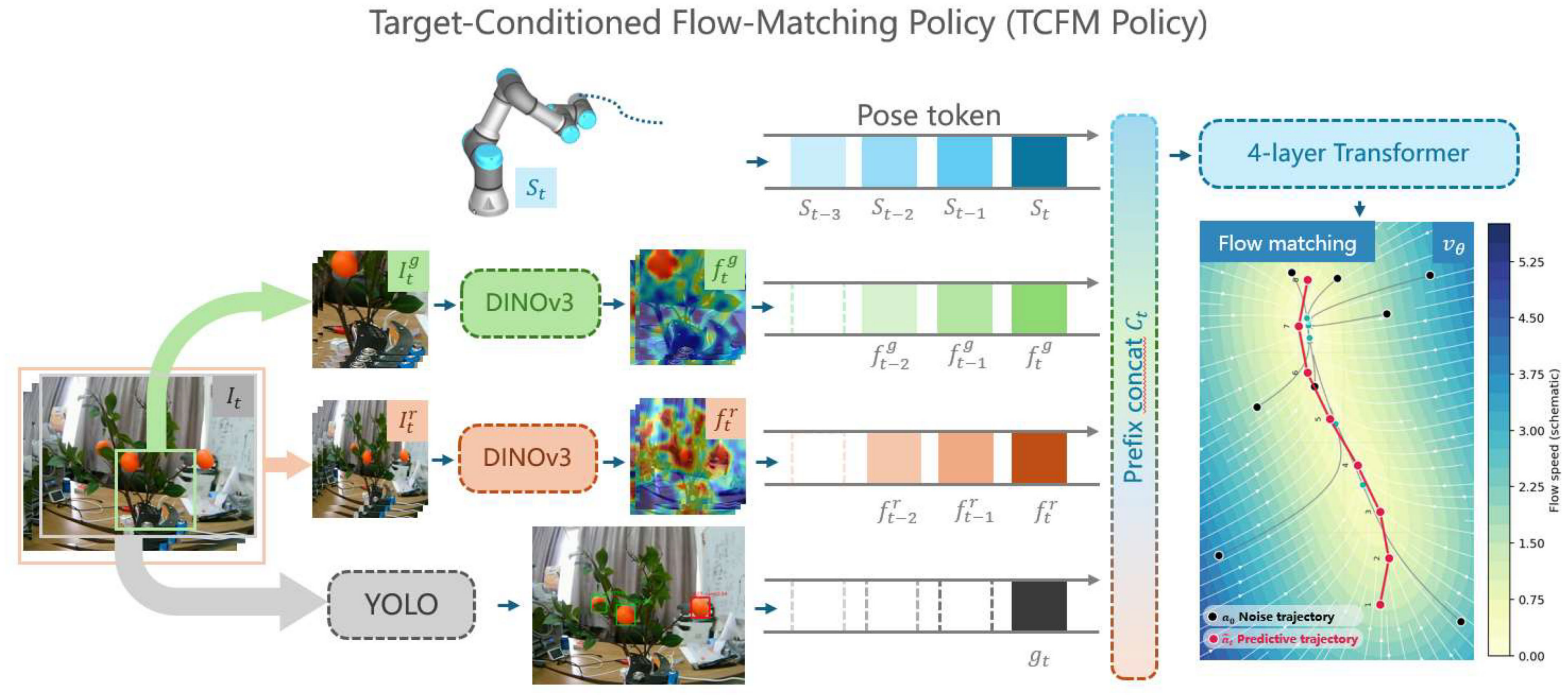
Overall framework of the TCFM Policy and the target-conditioned multi-step trajectory generation process.

The proposed TCFM Policy takes image observations, target-fruit geometric conditions, and end-effector state history as joint inputs and directly predicts future multi-step TCP approach trajectories toward a specified fruit. The overall framework is shown in **Figure 1**. The policy receives image history, state history, and target condition at time step *t*, extracts visual representations from both global and local views, and then generates a future trajectory chunk through a conditional flow-matching network:

1. Visual input: The first-person camera image from the robot end-effector is denoted by $I_{t}$. Given an image history of length $T_{I}$, the visual input sequence is

$$I_{t}=[I_{t-T_{I}+1},\ldots ,I_{t}]$$

2. Robot state input: The current 6D TCP pose relative to the robot base is denoted by $s_{t}\in {\mathbb{R}}^{6}$. Given a state history of length $T_{S}$, the state sequence is

$$S_{t}=[s_{t-T_{S}+1},\ldots ,s_{t}]$$

3. Target condition: The target fruit selected by the detection network is represented by

$${ɡ}_{t}=(x,y,w,h,\rho )$$

where x and y denote the target-box coordinates, w and h denote the box width and height, and ρ∈[0,1] is the detection confidence.

The TCFM policy is defined as.


$$\pi_{\theta }:(I_{t},S_{t},{ɡ}_{t})\mapsto {\hat{a}}_{t}$$

As shown in **Figure 1**, three image streams are used when $T_{I}=3$. The cropped ROI images are encoded by DINOv3 to produce ROI features $\left[f_{t-T_{I}+1}^{r},\ldots ,f_{t}^{r}\right]$, while resized global images are encoded by the same backbone to produce global features $\left[f_{t-T_{I}+1}^{ɡ},\ldots ,f_{t}^{ɡ}\right]$. These two sets of visual features are fused as.


$$F_{t}^{v}=\phi_{v}([f_{t-T_{I}+1}^{ɡ},\ldots ,f_{t}^{ɡ}],[f_{t-T_{I}+1}^{r},\ldots ,f_{t}^{r}])$$

Here, $\phi_{v}(\cdot )$ denotes the feature-fusion function. A third image stream uses only the current image $I_{t}$, from which a trained YOLOv11 detector extracts the target condition $g_{t}$. The visual feature, state history, and target condition are then combined to form the policy condition vector.


$$c_{t}=\phi_{c}(F_{t}^{v},S_{t},{ɡ}_{t})$$

This condition vector is fed into the flow-matching network to generate the future trajectory sequence.


$${\hat{a}}_{t}=FM_{\theta }(c_{t})$$

$${\hat{a}}_{t}=[{\hat{s}}_{t+1},\ldots ,{\hat{s}}_{t+H}]$$

where ${\hat{s}}_{t+\delta }\in {\mathbb{R}}^{6}$ denotes the predicted TCP state at a future step.

### 2.2 Data preparation and sample design for citrus harvesting

#### 2.2.1 Trajectory acquisition and smooth reconstruction based on VR teaching

Demonstration learning and imitation learning have started to show practical potential in agricultural harvesting tasks. Existing studies indicate that, in open-field and greenhouse environments, policies learned from expert demonstrations can partially bypass the difficulty of precise system modeling by directly converting local approach experience and end-effector adjustment patterns into executable control behavior (van de Ven et al., 2025; Kim et al., 2025). In addition, a review of selective harvesting robotics has pointed out that one of the central bottlenecks in agricultural harvesting lies in the large environmental variability and the finely structured final approach stage, which is difficult to parameterize completely (Kootstra et al., 2021). Based on this motivation, this study use VR teleoperation to construct an expert demonstration dataset so as to preserve temporal control patterns related to target approach, local obstacle avoidance, and final end-effector alignment.

**Figure 2:**
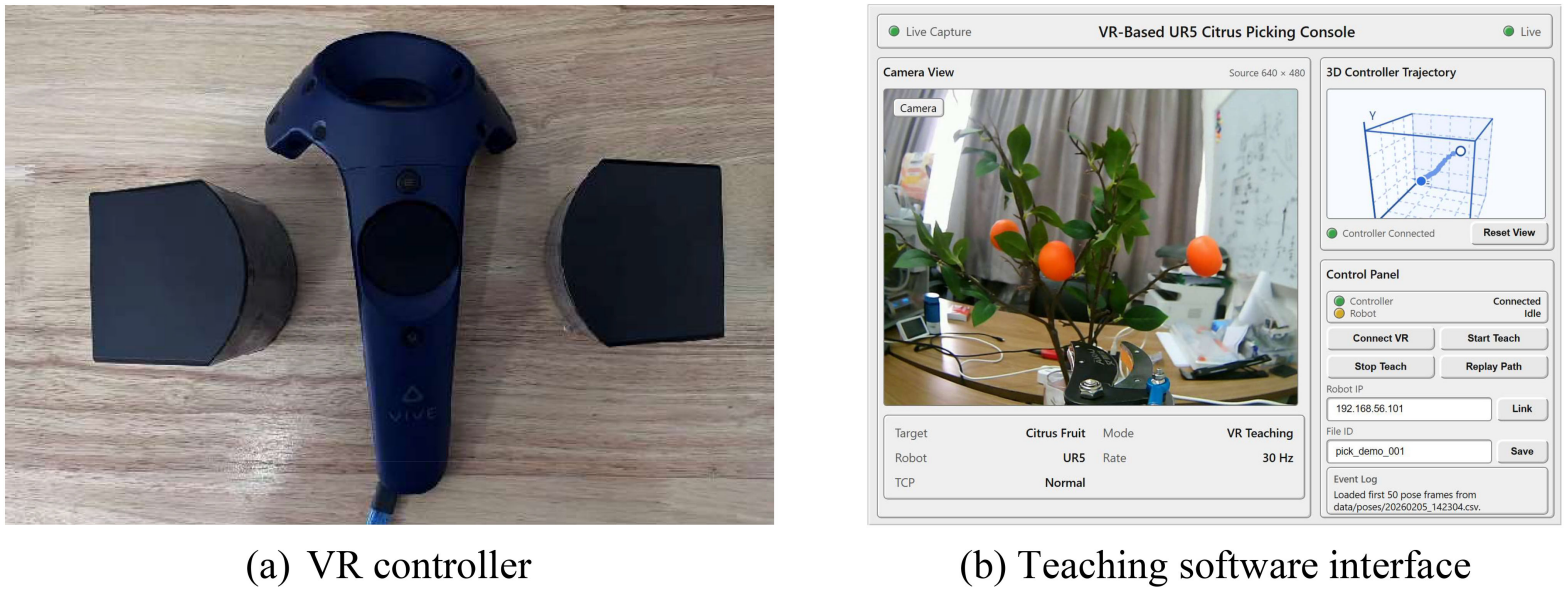
VR-based citrus harvesting demonstration and data acquisition platform. **(a)** VR controller used for harvesting demonstration in the virtual environment. **(b)** Teaching software interface for recording demonstrations and acquiring operation data.

The demonstration platform uses a UR5 manipulator as the execution arm. The operator controls the end-effector through a VR controller in real time and guides it toward the target fruit until it reaches the picking point with an appropriate pose. Compared with a hard-coded pipeline, this approach records human approach strategies and local adjustment behaviors more naturally in cluttered foliage environments. The VR controller and upper-computer interface are shown in **Figure 2**. Under indoor dual-base-station positioning, the VR controller provides sub-centimeter spatial accuracy, which is sufficient for fine end-effector pose control during harvesting. Through this platform, visual observations, robot states, and corresponding expert trajectories are recorded synchronously to support subsequent behavior cloning and multi-step trajectory generation.

During demonstration, the initial pose of the VR controller is used as the reference origin. The relative pose increment of the controller is computed in real time and mapped to the target TCP pose variation of the robot. Let $\text{Δ}p_{t}\in {\mathbb{R}}^{6}$ be the pose increment of the VR controller at time *t*. The target control state is defined as:


$$s_{t}^{cmd}=s_{t}+\text{Δ}p_{t}$$

Here, $s_{t}^{cmd}$ denotes the target TCP state of the manipulator. This mapping enables synchronized motion between the VR controller and the robot end-effector, allowing the operator to demonstrate the harvesting process intuitively.

However, if the raw demonstration stream is used directly for training, the trajectory often contains hand jitter, local irregular disturbances, and a distribution mismatch between the human-demonstrated trajectory and the physically executable robot trajectory. These factors reduce sample quality and affect policy-learning stability. To address this issue, we adopt a data-construction pipeline based on low-frequency sampling, trajectory smoothing, and high-frequency replay. First, during manual teaching, the executed manipulator trajectory is sampled at 3 Hz, yielding a TCP state sequence:


$$\{s_{1},s_{2},\ldots ,s_{N}\},s_{i}\in {\mathbb{R}}^{6}$$

On this basis, a spline function is used to fit the discrete trajectory points with weighted smoothing, resulting in a continuous trajectory function:


$$\tilde{s}(\lambda )=f(\lambda ),\lambda \in [0,1]$$

Here, $\tilde{s}(\lambda )$ denotes the reconstructed continuous TCP trajectory and f(λ) denotes the smoothing function. To balance trajectory smoothness and terminal precision, a weighted fitting objective is introduced:


$$L_{fit}=\sum_{i=1}^{N}\omega_{i}∥f(\lambda_{i})-s_{i}{∥}_{2}^{2}$$

Here, $\omega_{i}$ is the weight associated with the i-th sampled point. Since the final approach stage is more sensitive to pose accuracy, larger weights are assigned to the later portion of the trajectory so that the fitted trajectory remains close to the original demonstrated poses in the terminal region, thereby reducing noticeable deviation in the harvesting-critical stage while suppressing local jitter noise.

After the trajectory has been fitted, the manipulator returns to the initial pose and re-executes the task along the smoothed continuous trajectory. During this replay stage, TCP poses and visual images are collected at 10 Hz and used as the final training samples. Compared with direct use of the raw demonstration stream, this procedure improves trajectory smoothness while preserving the geometric characteristics and operational tendency of the original task.

#### 2.2.2 Dataset composition and target condition annotation

**Figure 3:**
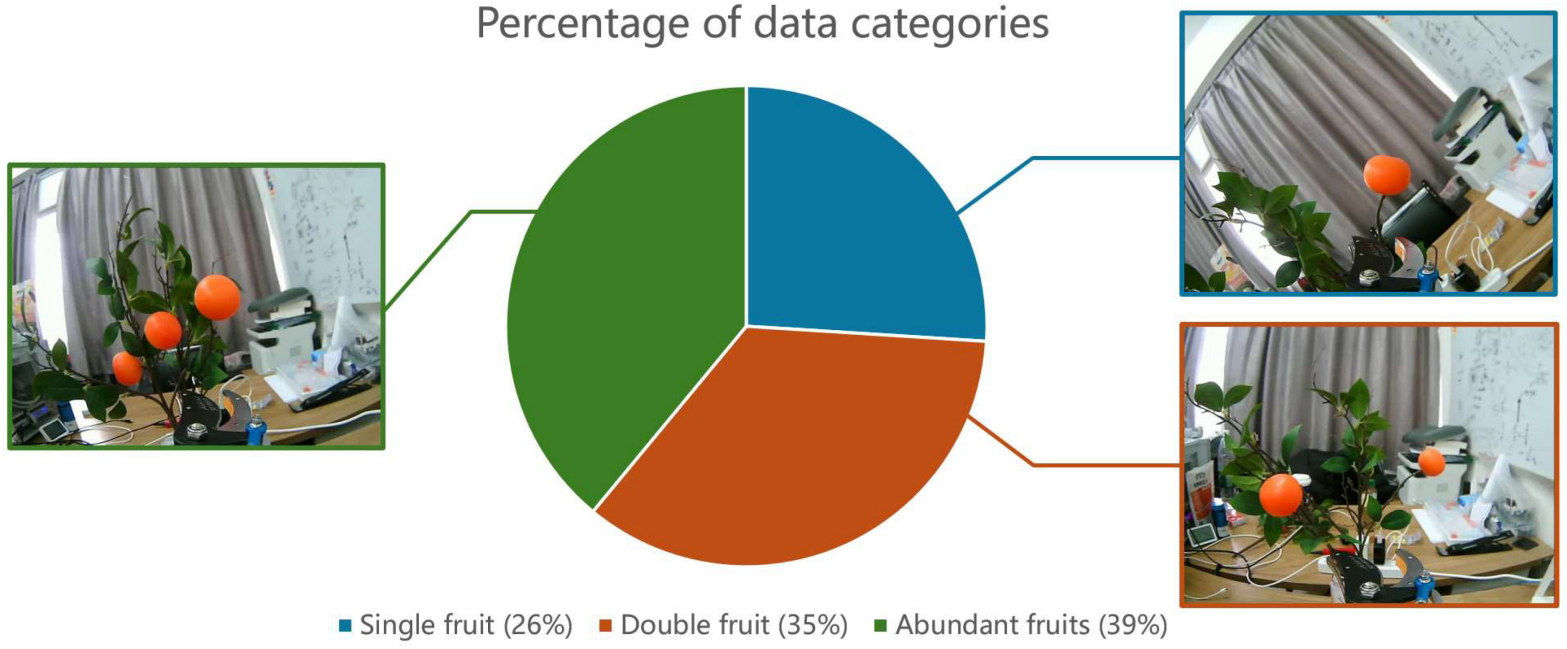
Distribution of scene categories in the citrus harvesting demonstration dataset.

In total, 160 real citrus harvesting demonstration episodes were collected. To cover common target-distribution patterns in harvesting and to improve the adaptability of the policy to complex scenes, the dataset is divided into three categories according to scene complexity: target picking in single-fruit scenes, specified single-target picking in dual-fruit scenes, and specified single-target picking in multi-fruit scenes. This design ensures that the training data include varying levels of difficulty, such as clear targets, local occlusion, and multi-target interference, thereby providing a basis for evaluating target consistency and generalization in cluttered foliage environments. The distribution of the three categories is shown in **Figure 3**.

**Figure 4:**
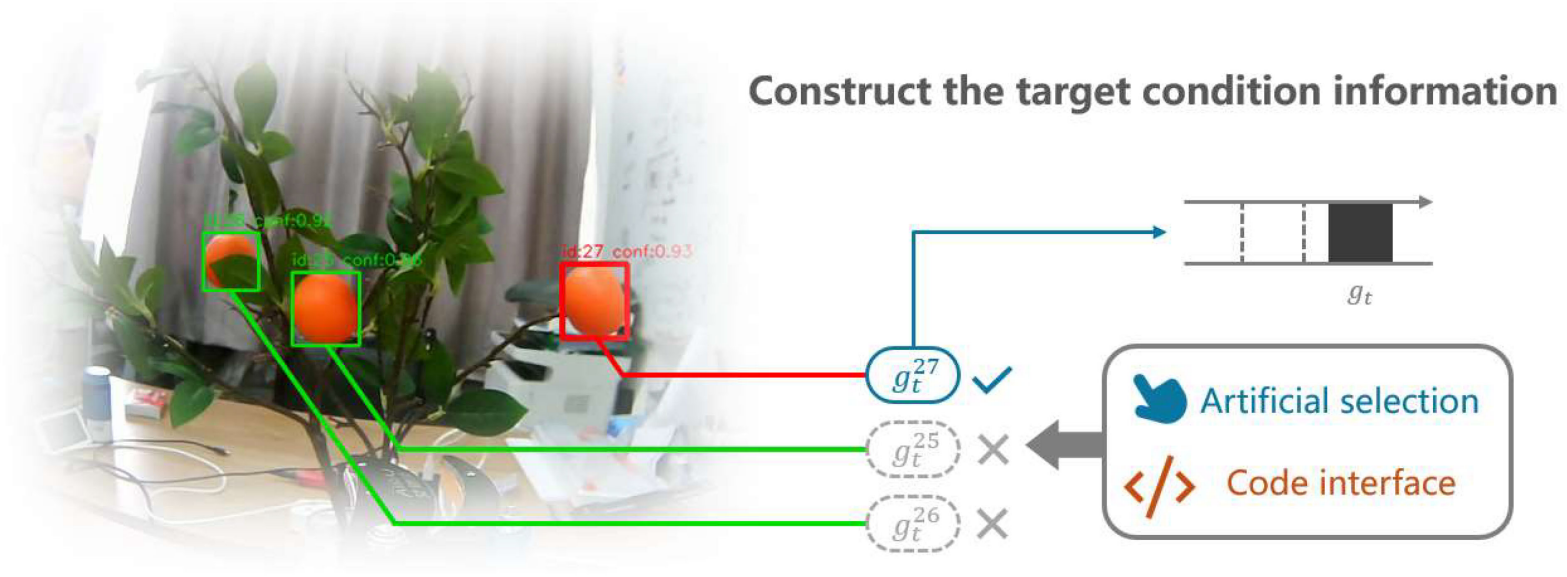
Construction of target-condition information and target-fruit annotation workflow.

To construct the target-condition information required for policy learning, an in-house trained YOLO detector is used for offline recognition of the demonstration image sequences. As shown in **Figure 4**, a citrus detection model is first trained on manually annotated images. The trained detector is then applied frame by frame to the demonstration videos to obtain candidate fruit bounding boxes and their geometric attributes. Finally, each demonstrated harvesting trajectory is manually matched to its corresponding target fruit based on the actual demonstration process and trajectory, establishing a correspondence among visual observation, target object, and action trajectory for each sample and generating the target-condition input used for policy learning.

#### 2.2.3 Target-oriented visual data augmentation

Under limited demonstration data, behavior-cloning policies tend to rely excessively on non-essential visual cues such as background textures, illumination distribution, and fruit appearance, which weakens attention to target spatial relationships and local end-effector operation cues and reduces robustness in complex scenes. To mitigate this issue, this study introduce a target-oriented visual augmentation strategy during training, applied mainly to the global-image branch, so as to intentionally reduce the model’s dependence on detailed global appearance.

Specifically, let the original global image be I and the augmented image be $\tilde{I}$. During training, augmentation is triggered only on the global branch with probability $p_{aug}=0.12$. Once triggered, one of three operations is selected at random: Gaussian blur, noise-added blur, or blackout. Gaussian blur is defined as.


$$\tilde{I}=G_{\sigma }*I$$

where $G_{\sigma }$ denotes the Gaussian kernel and * denotes convolution. Noise-added blur first perturbs the image with Gaussian noise and then applies blur:


$$\left\{ \begin{array}{c} I'=clip(I+\in ,0,L),\in \sim N(0,\sigma_{n}^{2})\\ \tilde{I}=G_{\sigma_{b}}*I' \end{array}\right.$$

Here, L is the upper bound of the pixel intensity. Blackout sets the entire global image to zero:


$$\tilde{I}(x,y,c)=0,\forall (x,y,c)$$

**Figure 5:**
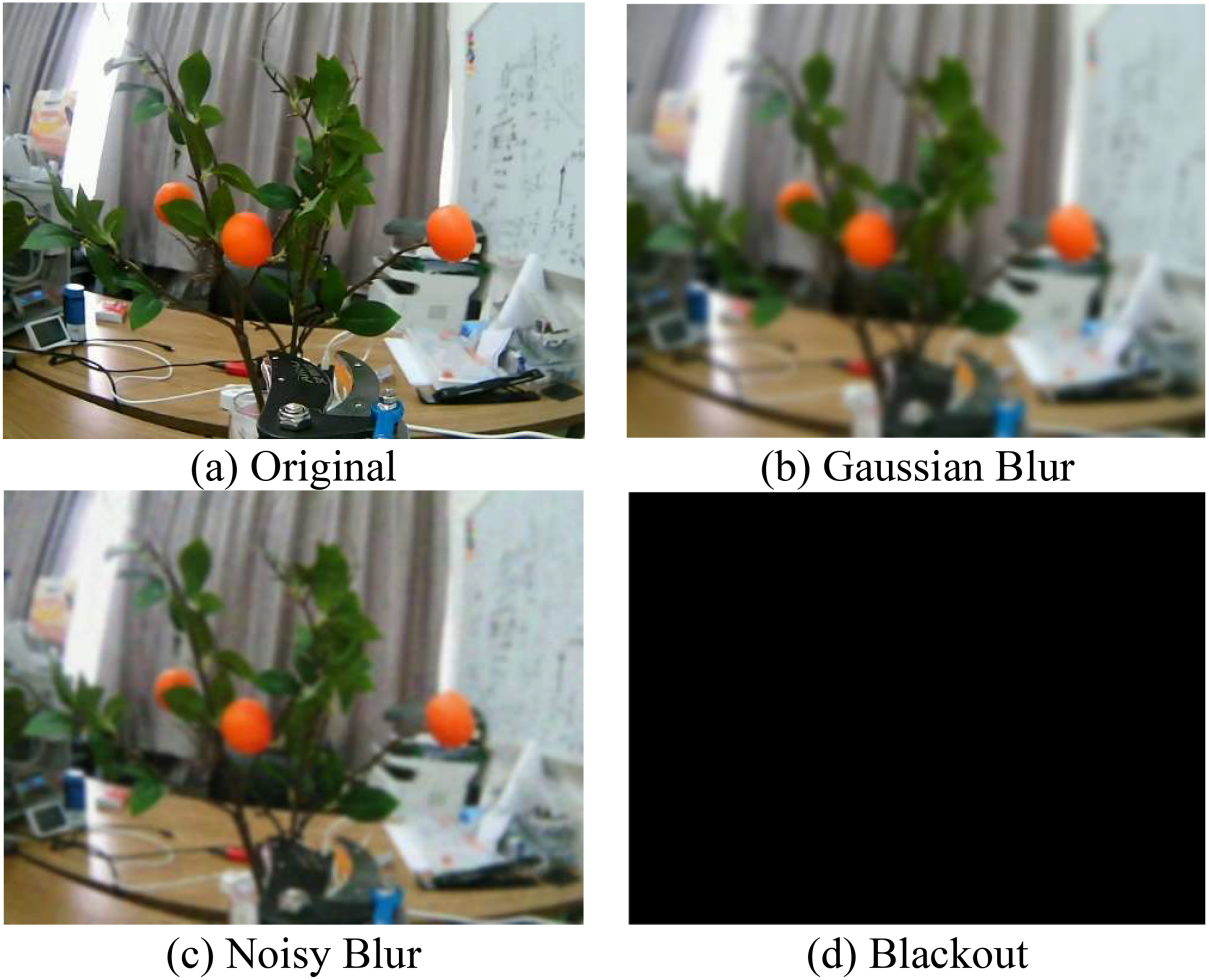
Examples of target-oriented visual data augmentation. **(a)** Original. **(b)** Gaussian blur. **(c)** Noisy blur. **(d)** Blackout.

Through these augmentations, the model cannot stably depend on fine-grained appearance information in the global image during training and is forced to make decisions by relying more on local ROI information and target geometric conditions. This improves learning stability and visual robustness in complex citrus-harvesting scenes. **Figure 5** shows examples of the original image, Gaussian blur, noise-added blur, and blackout.

### 2.3 Target-conditioned dual-branch visual representation

Action decisions in citrus harvesting depend on both scene-level context and fine-grained local details. Global images preserve the spatial relationship among the target fruit, surrounding foliage, neighboring fruits, and the end-effector within the whole scene. By contrast, local ROI images centered on the end-effector workspace preserve fine texture and local geometry in the stem neighborhood, branch-contact region, and tool vicinity. Either view alone is insufficient: global images may dilute the target region in background clutter, whereas local crops may lose the broader scene relationship needed for motion planning.

**Figure 6:**
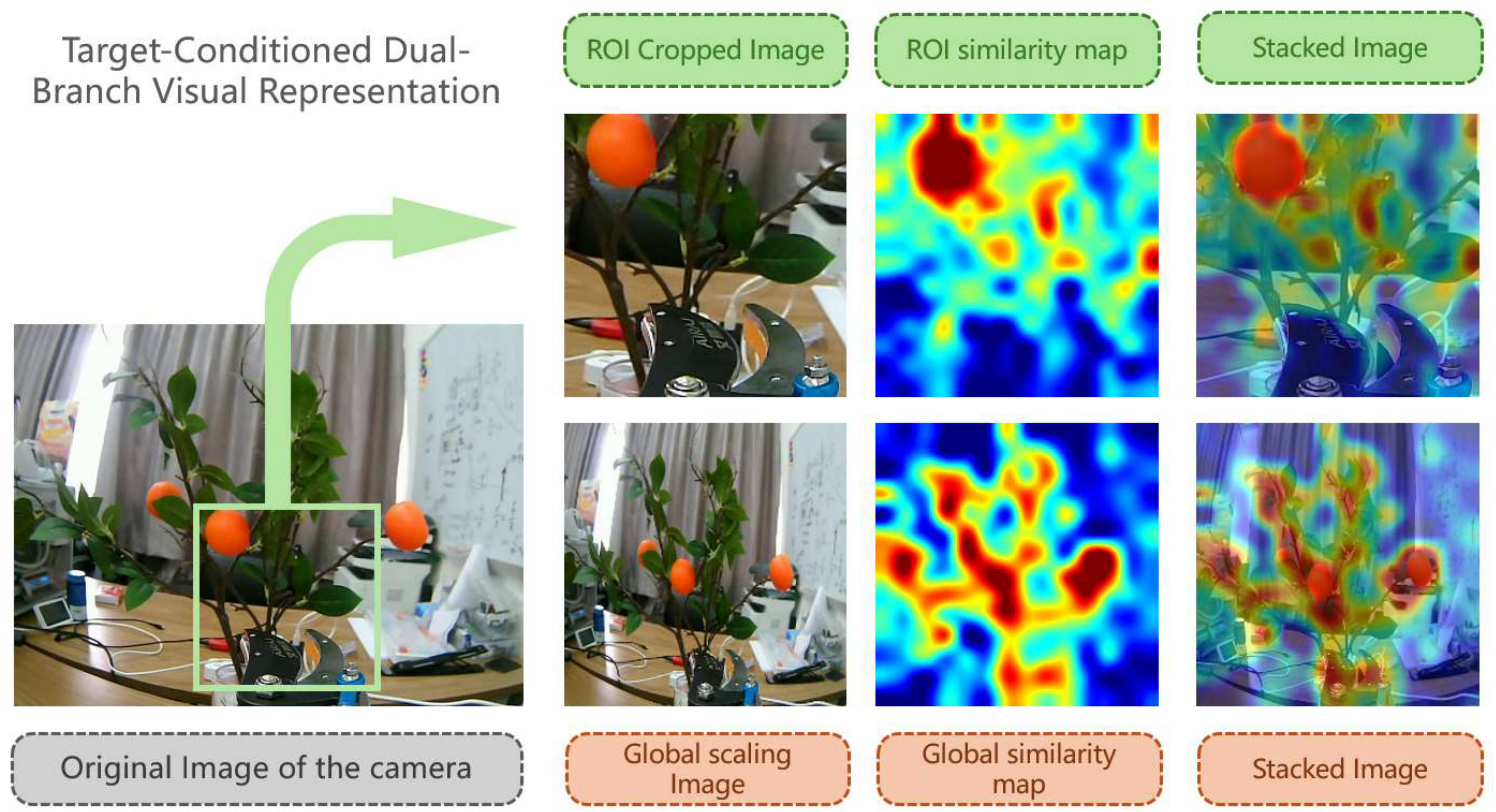
Construction of the target-conditioned dual-branch visual representation.

To balance these two requirements, a dual-branch visual representation is constructed by jointly encoding global-image history and ROI-image history with a shared DINOv3 backbone. Large-scale self-supervised pretrained models can learn transferable visual representations without task-specific labels and show strong stability across scenes, textures, and local visual details (Huang et al., 2025). The encoder is frozen and only the downstream policy network is trained. This design reduces overfitting under small-sample conditions while retaining transferable visual features learned from large-scale self-supervised pretraining. As shown in **Figure 6**, the resulting features highlight fruits, branches, and the end-effector neighborhood, indicating that the representation captures task-relevant scene structure effectively.

### 2.4 Flow-matching-based multi-step trajectory prediction

Because the final approach stage of citrus harvesting often admits multiple feasible paths, deterministic one-step regression or autoregressive prediction tends to accumulate errors and cannot adequately capture multi-modal action distributions. This study therefore model multi-step trajectory generation as a conditional flow-matching problem: given the condition $c_{t}$, the model learns a conditional velocity field that continuously transports a noisy trajectory distribution to the real trajectory distribution. This formulation predicts an entire trajectory chunk rather than a single future step, thereby improving the representation of multi-solution approach trajectories while preserving motion continuity.

Recent progress in generative policy learning supports this design choice. Diffusion-based and flow-based methods have shown advantages in modeling multi-modal continuous action distributions in robot policy learning (Rouxel et al., 2024; Chi et al., 2025). Based on these observations, we formulate future TCP trajectory prediction under target conditions as a conditional flow-matching problem to jointly address trajectory consistency, multi-modality, and inference efficiency.

To generate a trajectory, a trajectory state $x_{\tau }$ parameterized by a continuous flow-time variable τ∈[0,1] is introduced here, and the conditional velocity field is defined as.


$$\frac{dx_{\tau }}{d\tau }=v_{\theta }(x_{\tau },\tau ,c_{t})$$

During inference, the trajectory state is initialized from Gaussian noise and is gradually transported from the noise distribution toward the real trajectory manifold:


$$x_{\tau =1}\sim N(0,I),{\hat{a}}_{t}=x_{\tau =0}$$

**Figure 7:**
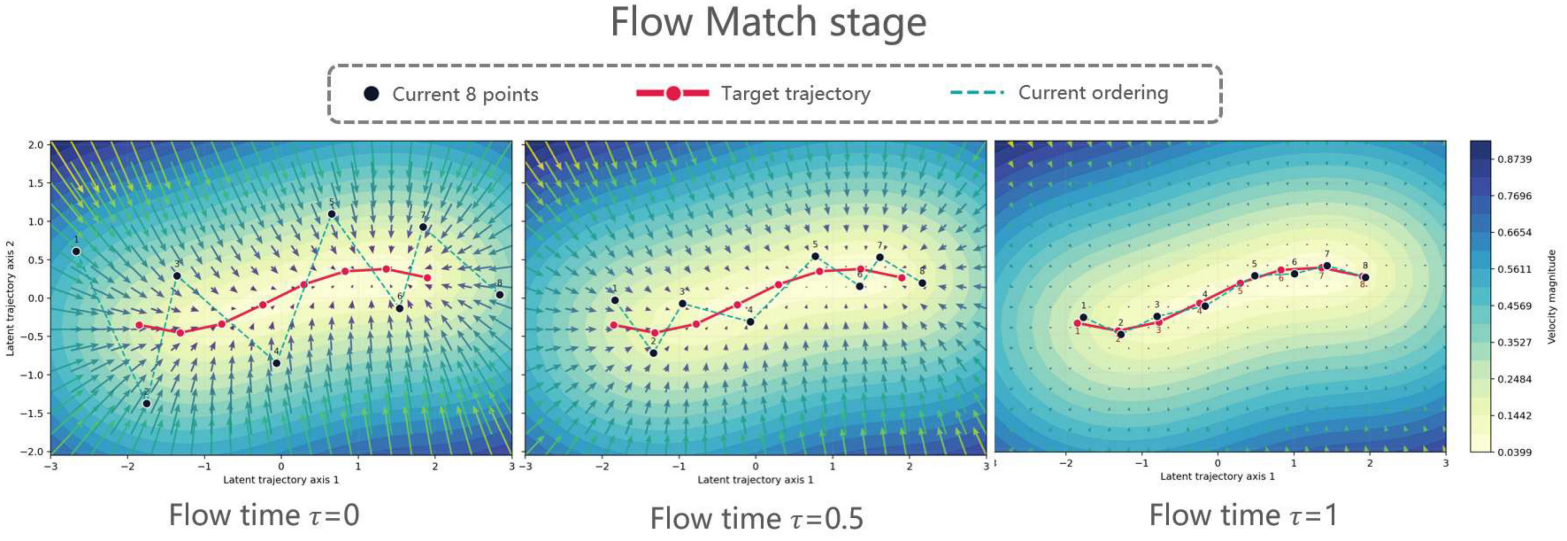
Illustration of multi-step trajectory generation with conditional flow matching.


In practice, the continuous flow process is solved with Euler discretization:


$$x_{\tau_{k+1}}=x_{\tau_{k}}+\text{Δ}\tau v_{\theta }(x_{\tau_{k}},\tau_{k},c_{t}),\text{Δ}\tau =-\frac{1}{N}$$

where N is the number of sampling steps. After iterative updates, the model outputs the predicted future H-step TCP trajectory (show in **Figure 7**):

$${\hat{a}}_{t}=[{\hat{s}}_{t+1},{\hat{s}}_{t+2},\ldots ,{\hat{s}}_{t+H}],{\hat{s}}_{t+\delta }\in {\mathbb{R}}^{6}$$

From a modeling perspective, the proposed method does not predict future actions point by point in an autoregressive manner. Instead, it directly learns the overall transport direction of the complete future trajectory in flow space under the condition $c_{t}$, thereby enabling parallel generation of multi-step trajectories. The final algorithm flow is shown in **Algorithm 1**.

Algorithm 1

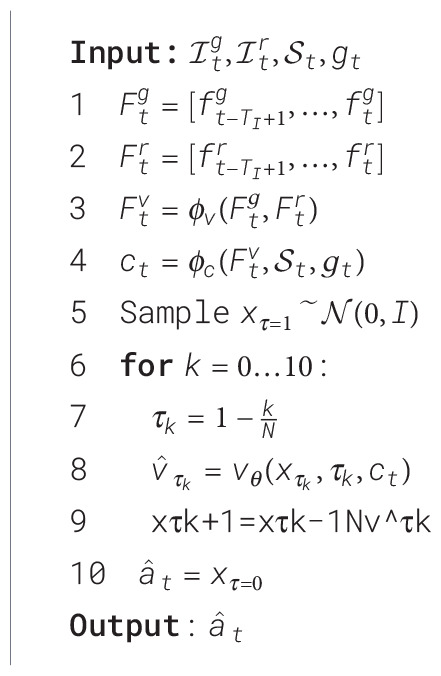

### 2.5 Network training and implementation details

This section describes how the proposed policy network is trained and implemented, with emphasis on the supervision target of conditional flow matching, the construction of intermediate trajectory states, and the main hyperparameter settings. The goal is to clarify how the model learns to predict future TCP trajectories from conditional inputs.

The proposed network is trained using the conditional flow-matching objective. Let the real future trajectory be.


$$a_{t}=[s_{t+1},\ldots ,s_{t+H}]\in {\mathbb{R}}^{H\times D_{a}}$$

where H is the prediction horizon and $D_{a}=6$ is the action dimension corresponding to the 6D TCP pose. During training, Gaussian noise is first sampled:


∈~N(0,I)

The flow-time variable is then sampled from a Beta distribution:


τ~Beta(α,β)

An intermediate trajectory state is constructed by linear interpolation:


$$x_{\tau }=\tau \in +(1-\tau )a_{t}$$

The corresponding target velocity field is defined as.


$$u_{\tau }=\in -a_{t}$$

Given the condition representation $c_{t}=\phi_{c}(F_{t}^{v},S_{t},g_{t})$, the network predicts the conditional velocity field.


$${\hat{v}}_{\tau }=v_{\theta }(x_{\tau },\tau ,c_{t})$$

To make the predicted velocity field approach the target velocity field, this study use an element-wise $L_{1}$ flow-matching loss:


$$L_{\text{FM}}=\frac{1}{\sum_{h=1}^{H}m_{h}}\sum_{h=1}^{H}\sum_{d=1}^{D_{a}}m_{h}|{\hat{v}}_{\tau ,h,d}-u_{\tau ,h,d}|$$

Here, $m_{h}\in \{0,1\}$ is the validity mask of the h-th future step. This mask is used to prevent padded samples near trajectory boundaries from interfering with training. The final algorithm flow is shown in **Algorithm 2**.

Algorithm 2

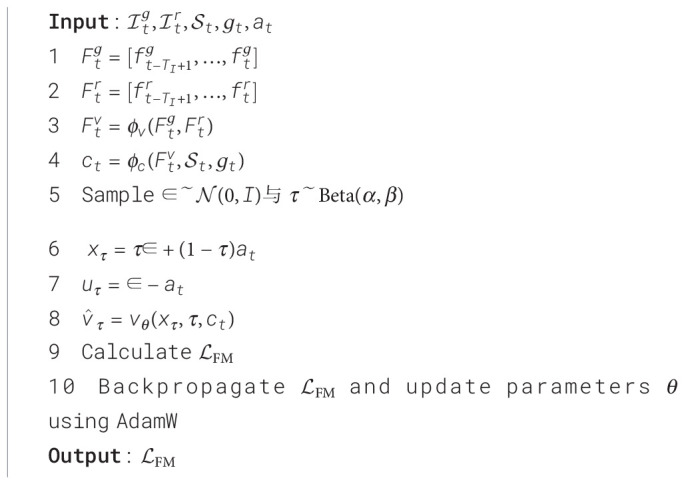

In the current implementation, the image-history length is set to $T_{I}=3$, the state-history length is set to $T_{S}=4$, and the prediction horizon is set to H=8. Both the global-image branch and the ROI-image branch use DINOv3 as the visual encoder. The flow-matching backbone is a lightweight Transformer with hidden dimension 256, 4 Transformer blocks, and 4 attention heads. During inference, Euler discretization with N=10 sampling steps is used in the default configuration of the proposed TCFM Policy. During training, the flow-time variable is sampled from a Beta distribution with α=1.5 and β=1.0. The network parameters are optimized by backpropagation using AdamW.

## 3 Experiments

### 3.1 Experimental setup and evaluation metrics

The experimental dataset contains 160 valid demonstration episodes. To avoid information leakage, the dataset is split at the episode level rather than at the frame level. Specifically, 144 episodes are used for training and 16 episodes are used for testing, with no separate validation set. The main training settings are summarized in **Table 1**.

**Table 1:** Dataset split and main training settings.

Item	Setting
Number of valid demonstration episodes	160
Training/validation/test episodes	144/0/16
Single-fruit/dual-fruit/multi-fruit samples	42/56/62
Image-history length $T_{I}$	3
State-history length $T_{S}$	4
Prediction horizon H	8
Batch size	16
Learning rate	0.0001
Epochs	20
Optimizer	AdamW
Random seed	42

At time t, the TCFM Policy takes three types of input: image history $I_{t}$, state history $S_{t}$, and target condition $g_{t}$. The image history consists of $T_{I}=3$ global images and their corresponding ROI images, and the state history has length $T_{S}=4$. The output is the future H=8-step TCP trajectory ${\hat{a}}_{t}=[{\hat{s}}_{t+1},{\hat{s}}_{t+2},\ldots ,{\hat{s}}_{t+H}]$. The network is trained with AdamW, a batch size of 16, a learning rate of $1\times 10^{-4}$, 20 training epochs, and a random seed of 42. During inference, Euler discretization with N=10 sampling steps is used, and the flow-time variable follows τ~Beta(α,β) with α=1.5 and β=1.0During online deployment, the policy is executed in a receding-horizon manner. At each inference cycle, the latest image observations, target condition, and TCP state history are updated, and the policy predicts an 8-step future TCP trajectory chunk. In the online experiments reported in this paper, the predicted 8-step chunk is executed before the next inference cycle is triggered. Therefore, the system still operates as a perception-inference-execution loop at the TCP trajectory level rather than as a one-shot trajectory rollout over the entire task. In principle, the number of executed steps can be reduced under stronger environmental disturbances to obtain a tighter real-time feedback loop. However, in the current experiments, no target-loss event was observed during execution, and such a shorter execution window was therefore not activated.

**Figure 8:**
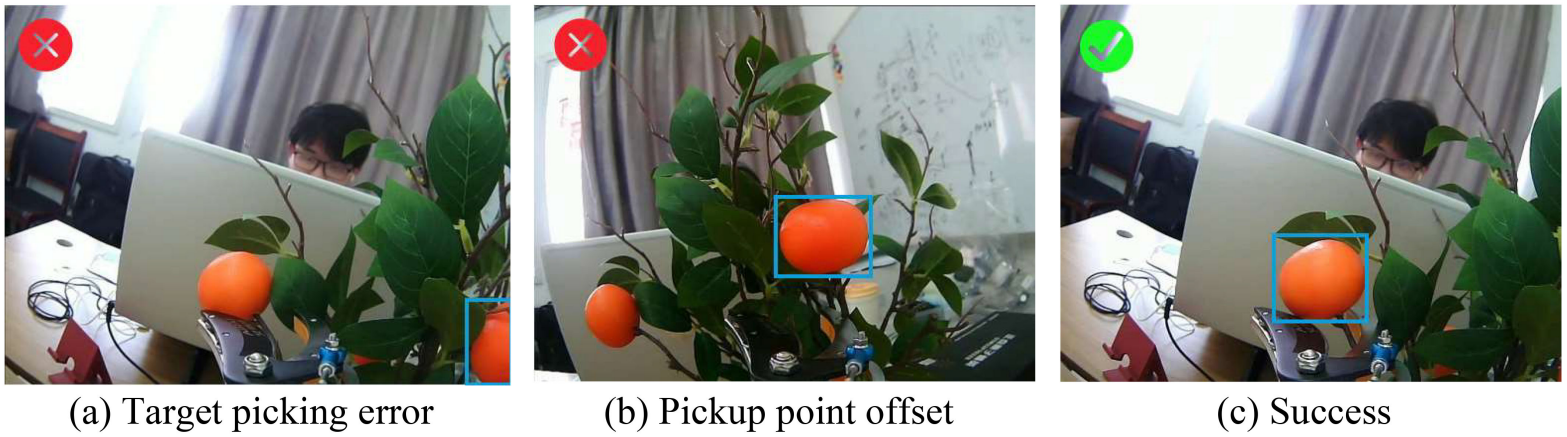
Example of result judgment for multi-fruit scenarios during harvesting. **(a)** Target-picking error. **(b)** Picking-point offset. **(c)** Success.


To evaluate the proposed method from both task completion and trajectory-fitting perspectives, this study use online task metrics and offline error metrics. In online evaluation, a trial is counted as successful only if the robot consistently approaches the specified target fruit throughout execution and finally reaches the valid picking point associated with that target (**Figure 8C**). If the robot switches targets or deviates to a non-specified fruit during execution, the outcome is counted as a target-picking error (**Figure 8A**). If the robot approaches the target fruit but fails to reach the valid picking point accurately, the outcome is counted as a picking-point offset (**Figure 8B**). Let the total number of test trials be M, and let the numbers of successful trials, target-picking errors, and picking-point offsets be $M_{succ}$, $M_{err}$, and $M_{off}$, respectively. The three online metrics are defined as.

$$\left\{ \begin{array}{c} \text{SuccessRate}=\frac{M_{succ}}{M},\\ \text{TagetPickingErrorRate}=\frac{M_{err}}{M},\\ \text{Picking}-\text{PointOffsetRate}=\frac{M_{off}}{M}. \end{array}\right.$$

The offline evaluation measures the average deviation between the predicted trajectory ${\hat{a}}_{t}$ and the real future trajectory $a_{t}\in {\mathbb{R}}^{H\times D_{a}}$. Suppose the test set contains N evaluation samples, and for the i-th sample at time t, the real and predicted future trajectories are denoted by $a_{t}^{\left(i\right)}$ and ${\hat{a}}_{t}^{\left(i\right)}$, respectively. The overall mean absolute error is defined as.


$${\text{MAE}}_{\text{all}}=\frac{1}{NHD_{a}}\sum_{i=1}^{N}\sum_{h=1}^{H}\sum_{d=1}^{D_{a}}|{\hat{a}}_{t,h,d}^{\left(i\right)}-a_{t,h,d}^{\left(i\right)}|$$

Let the first three dimensions of the trajectory be the position component $p_{t,h}^{\left(i\right)}\in {\mathbb{R}}^{3}$ and the last three dimensions be the orientation component $r_{t,h}^{\left(i\right)}\in {\mathbb{R}}^{3}$, with predictions ${\hat{p}}_{t,h}^{\left(i\right)}$ and ${\hat{r}}_{t,h}^{\left(i\right)}$, respectively. The position and orientation errors are defined as.


$$\left\{ \begin{array}{c} MA{\text{E}}_{xyz}=\frac{1}{3NH}\sum_{i=1}^{N}\sum_{h=1}^{H}∥{\hat{p}}_{t,h}^{\left(i\right)}-p_{t,h}^{\left(i\right)}{∥}_{1}\\ MA{\text{E}}_{rpy}=\frac{1}{3NH}\sum_{i=1}^{N}\sum_{h=1}^{H}∥{\hat{r}}_{t,h}^{\left(i\right)}-r_{t,h}^{\left(i\right)}{∥}_{1} \end{array}\right.$$

To reflect end-effector spatial deviation more directly, we also compute the mean Euclidean position error:


$$\text{L}2_{\text{pos}}=\frac{1}{NH}\sum_{i=1}^{N}\sum_{h=1}^{H}∥{\hat{p}}_{t,h}^{\left(i\right)}-p_{t,h}^{\left(i\right)}{∥}_{2}$$

For the k-th future prediction step, the step-wise MAE is defined as.


$${\text{MAE}}_{k}=\frac{1}{ND_{a}}\sum_{i=1}^{N}\sum_{d=1}^{D_{a}}|{\hat{a}}_{t,k,d}^{\left(i\right)}-a_{t,k,d}^{\left(i\right)}|,k=1,2,\ldots ,H.$$

Together, these metrics evaluate the proposed method from the perspectives of target consistency, execution effectiveness, and trajectory-fitting ability, thereby providing a more complete assessment of TCFM Policy in multi-fruit citrus harvesting.

Trials in which the predicted TCP endpoint could not be executed because inverse kinematics failed during robot deployment, for example due to joint-solution ambiguity or the predicted pose approaching joint-limit configurations, were excluded from both the success and failure counts and were not included in the reported statistics. These cases were treated as uncounted trials because the present study focuses on policy-level target-consistent TCP approach generation rather than joint-space feasibility analysis.

### 3.2 Target-conditioned path generation in multi-fruit scenes

**Figure 9:**
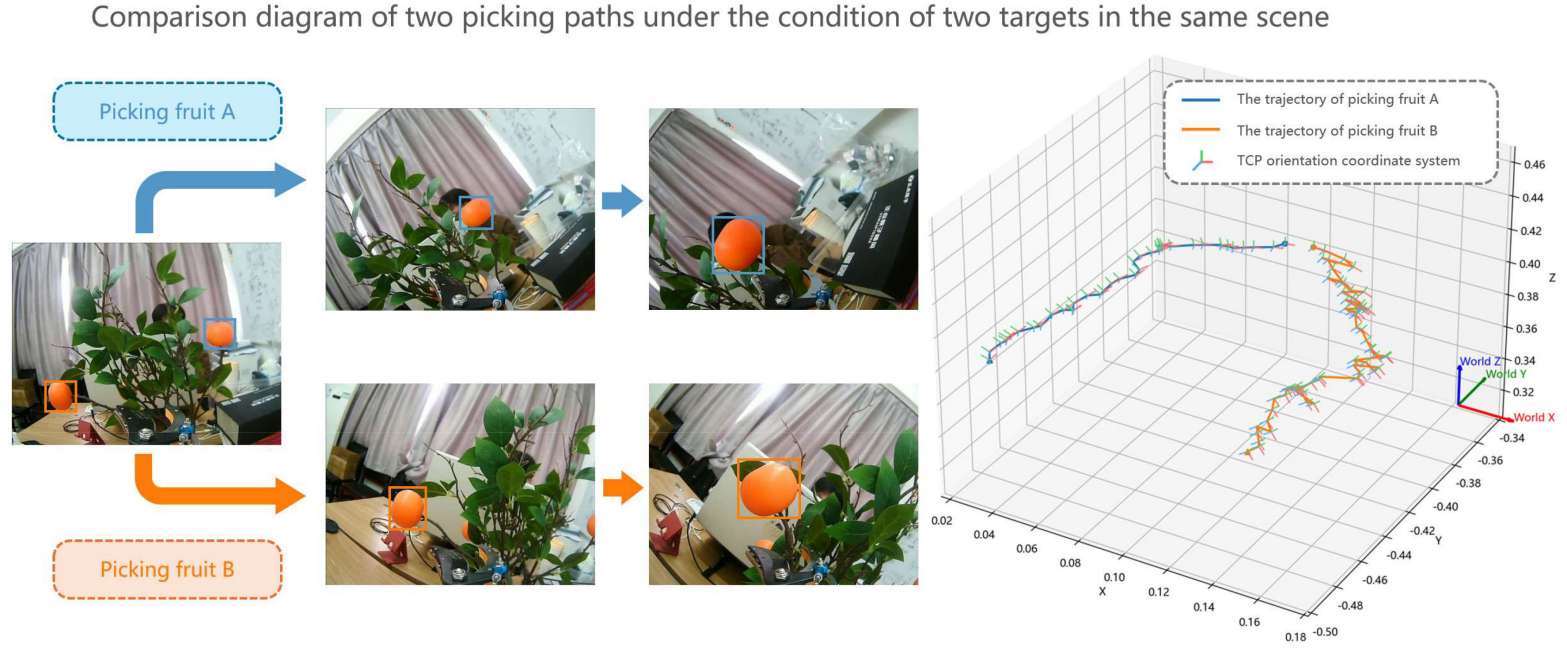
Generation results of picking paths corresponding to different target conditions in the same scene.

**Figure 9** presents the target-conditioned trajectory generation results for two different target fruits in the same multi-fruit scene. When the externally specified target is switched from fruit A to fruit B, while the scene observation $I_{t}$ and state history $S_{t}$ remain nearly unchanged, the TCFM Policy generates two clearly different approach trajectories by changing only the target condition $g_{t}$. The two trajectories finally converge to the picking regions of the corresponding targets. This result indicates that the target condition is not merely an auxiliary label but directly modulates the condition representation $c_{t}=\phi_{c}(F_{t}^{v},S_{t},g_{t})$ and hence affects the overall generation direction of the subsequent multi-step trajectory ${\hat{a}}_{t}$.

Further observation of the 3D trajectories in **Figure 9** shows that the two trajectories differ significantly in their approach directions toward the two fruits and exhibit almost no overlap near the targets. This phenomenon shows that the proposed target-conditioning mechanism can explicitly constrain the end-effector approach direction while preserving trajectory continuity and executability, thereby effectively mitigating target ambiguity in multi-target scenes. This capability is particularly important for agricultural harvesting, because adjacent fruits often have similar color and shape. A policy learned only through image-based regression tends to produce compromise or averaged actions, whereas the proposed method can generate differentiated approach paths stably under a specified target condition.

### 3.3 Ablation study in multi-fruit scenes

To verify the contribution of each component of the TCFM Policy in multi-fruit target-specified approach tasks, ablation experiments are conducted under low-light conditions. Four configurations are compared: the full model, the model without the ROI branch, the model without target-oriented augmentation, and the model without both the ROI branch and augmentation. Each method is tested in 50 target-specified picking trials, and the results are listed in **Table 2**. The full model achieves a success rate of 76%, with only 4% target-picking errors and 20% picking-point offsets, indicating balanced performance in both target maintenance and accurate final alignment.

**Table 2:** Online picking results of different model configurations in multi-fruit scenes.

Method	Number of tests	Success rate	Target picking error rate	Pickup point offset rate
TCFM Policy	50	76%	4%	20%
w/o ROI	50	68%	2%	30%
w/o Augmentation	50	46%	38%	16%
w/o ROI and Augmentation	50	44%	38%	18%

The ablation results show that the ROI branch mainly improves local alignment accuracy during the final approach stage. After removing the ROI branch, the success rate decreases from 76% to 68%, while the picking-point offset rate increases from 20% to 30%. This suggests that, although global images still provide approximate directional information about the target, they lack fine-grained perception of the stem neighborhood, foliage occlusion relationships, and the local structure of the end-effector workspace during the critical final approach. As a result, the final accuracy of reaching the picking point decreases. Therefore, the local visual cues provided by the ROI branch play a meaningful role in improving final alignment accuracy.

By contrast, target-oriented augmentation contributes more strongly to target consistency. After removing the augmentation module, the success rate drops sharply from 76% to 46%, while the target-picking error rate increases from 4% to 38%. When both the ROI branch and augmentation are removed, the success rate further decreases to 44%, and the target-picking error rate remains at a high level of 38%. These results indicate that, under limited demonstration data, the model tends to over-rely on background texture, illumination distribution, and fruit appearance if no mechanism is used to suppress dependence on global appearance. The random blur, noise-added blur, and blackout operations introduced in this study force the model to rely more on ROI-local information and target geometric conditions when constructing $F_{t}^{v}$ and $c_{t}$, thereby substantially reducing target loss and wrong-target picking.

Overall, the ROI branch and target-oriented augmentation are functionally complementary: the former mainly addresses accurate arrival at the picking point, whereas the latter mainly addresses correct target identification. Together with the explicit target-condition input, they form the key basis for stable target-consistent approach in multi-fruit scenes.

### 3.4 Offline trajectory error as supplementary evidence

To further analyze model performance from the trajectory-fitting perspective, this study compute ${\text{MAE}}_{\text{all}}$, ${\text{MAE}}_{xyz}$, ${\text{MAE}}_{rpy}$, and $\text{L}2_{\text{pos}}$ for different model configurations on 3,833 evaluation samples from the test set, as shown in **Table 3**. Overall, the numerical differences in offline error metrics are relatively small across all configurations. Specifically, $MAE_{all}$ ranges from 0.0315 to 0.0332, and ranges from 0.0237 to 0.0269. This indicates that all models can capture the overall trend of the real future trajectory $a_{t}$ to a certain extent, even when one module is removed.

**Table 3:** Offline trajectory error comparison of different model configurations on the test set.

Method	No. of Eval. Samples	Mean $MAE_{all}$	Mean $MAE_{xyz}$	Mean $MAE_{rpy}$	Mean end-effector position L2 error
TCFM Policy	3833	0.0332	0.0090	0.0573	0.0258
w/o Target condition	3833	0.0327	0.0094	0.0560	0.0268
w/o ROI	3833	0.0331	0.0089	0.0573	0.0257
w/o Augmentation	3833	0.0315	0.0084	0.0545	0.0237

However, the offline average error is not fully consistent with the online task success rate. Although the full model does not achieve the best value on every offline metric, it obtains the highest online picking success rate and the lowest target-picking error rate. This suggests that, in multi-target harvesting, average trajectory error alone is insufficient to fully reflect target consistency and task executability. In particular, when multiple feasible approach paths exist, the offline error may remain low as long as the overall trend of the predicted trajectory ${\hat{a}}_{t}$ is close to that of the real trajectory $a_{t}$. Yet if the model deviates from the specified target at a critical moment, the online execution result can still deteriorate substantially. Therefore, **Table 3** should be regarded as supplementary evidence reflecting how well different models fit the trajectory distribution, rather than as the sole criterion for judging policy quality.

**Table 4** further reports step-wise MAE for the future H=8 prediction steps. The error of all methods increases gradually with prediction horizon, from approximately 0.028-0.029 at step 1 to approximately 0.048-0.050 at step 8. This trend is consistent with the general error-accumulation behavior in multi-step trajectory prediction. By comparison, the full model keeps the error growth within a controllable range over longer horizons, indicating that conditional flow matching can preserve overall trajectory continuity while generating multiple future TCP steps in parallel. Combined with the online results, these findings suggest that the main advantage of the proposed method does not lie in reducing pointwise MAE alone, but in maintaining higher target consistency and execution stability under multi-target interference, local occlusion, and appearance disturbance.

**Table 4:** Step-wise trajectory prediction MAE of different model configurations on the test set.

Method	Inference steps	Step 1 MAE	Step 2 MAE	Step 3 MAE	Step 4 MAE	Step 5 MAE	Step 6 MAE	Step 7 MAE	Step 8 MAE
TCFM policy	3833	0.0290	0.0346	0.0391	0.0431	0.0467	0.0430	0.0467	0.0496
w/o target condition	3833	0.0280	0.0335	0.0384	0.0427	0.0469	0.0427	0.0469	0.0499
w/o ROI	3833	0.0289	0.0341	0.0383	0.0426	0.0460	0.0426	0.0459	0.0488
w/o augmentation	3833	0.0278	0.0328	0.0373	0.0410	0.0445	0.0410	0.0445	0.0476

### 3.5 Comparison with a target-conditioned diffusion policy baseline

To further position the proposed method against a recent generative visuomotor baseline, a Target-Conditioned Diffusion Policy (TCDP Policy) is implemented. The TCDP baseline shares the same dataset split, normalization statistics, dual-branch visual front-end, target condition encoding, state history, action horizon, sequence backbone, and training budget as the proposed TCFM Policy. The main difference lies in the trajectory-generation head and the corresponding inference procedure. In the main comparison, the TCFM Policy uses 10 flow integration steps during inference, whereas the TCDP Policy uses 50 denoising steps.

As shown in **Table 5**, real-robot target-specified approach experiments further show that the proposed TCFM Policy outperforms the TCDP baseline. Under 50 trials, TCFM Policy achieved a success rate of 76%, a target-picking error rate of 4%, and a picking-point offset rate of 20%, whereas TCDP Policy obtained 62%, 6%, and 32%, respectively. These results indicate that the flow-matching policy provides more stable target-consistent approach behavior under the same system setting.

**Table 5:** Real-robot closed-loop target-specified approach comparison between TCFM and TCDP.

Method	Number of tests	Success rate	Target picking error rate	Pickup point offset rate
TCFM Policy	50	76%	4%	20%
TCDP Policy	50	62%	6%	32%

Offline trajectory evaluation also shows that the proposed TCFM Policy yields lower trajectory error than the TCDP baseline under the same training setting. As reported in **Table 6**, on 3,833 held-out samples, TCFM achieves an MAE_all of 0.0332, compared with 0.0407 for TCDP. Similar improvements are also observed in MAE_xyz, MAE_rpy, and end-effector position L2 error.

**Table 6:** Offline trajectory error comparison between TCFM and TCDP on the held-out test set.

Method	No. of Eval. Samples	Mean $MAE_{all}$	Mean $MAE_{xyz}$	Mean $MAE_{rpy}$	Mean end-effector position L2 error
TCFM Policy	3833	0.0332	0.0090	0.0573	0.0258
TCDP Policy	3833	0.0408	0.0109	0.0706	0.0313

**Figure 10:**
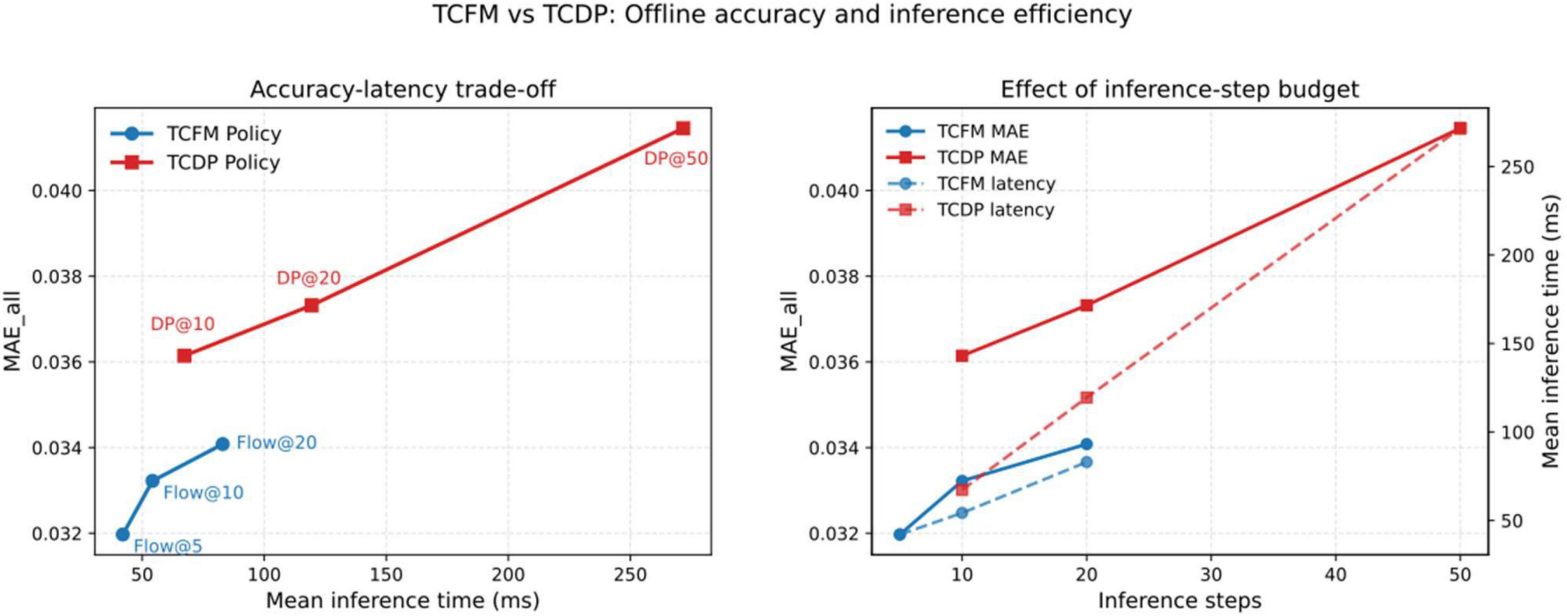
Offline accuracy and inference-efficiency comparison between TCFM and TCDP under different inference-step budgets.

To compare the two generative policies under different real-time budgets, additional experiments are conducted with different inference-step settings. For TCFM, the number of flow integration steps is set to 5, 10, and 20. For TCDP, the number of denoising steps is set to 10, 20, and 50. For each setting, both the held-out offline trajectory error and the average inference time measured over 50 repeated runs on the same evaluation sample are reported, as summarized in **Table 7** and **Figure 10**.

**Table 7:** Accuracy-latency trade-off of TCFM and TCDP under different inference-step budgets.

Method	Inference steps	Mean $MAE_{all}$	Mean $MAE_{xyz}$	Mean $MAE_{rpy}$	End-effector position L2 error	Mean inference time (ms)	Inference frequency (Hz)
TCFM Policy	5	0.0320	0.0085	0.0555	0.0246	42.071	23.769
TCFM Policy	10	0.0332	0.0090	0.0573	0.0258	54.247	18.434
TCFM Policy	20	0.0341	0.0094	0.0587	0.0268	82.993	12.049
TCDP Policy	10	0.0361	0.0093	0.0630	0.0272	67.292	14.861

As shown in **Figure 10**, the left panel compares the accuracy-latency trade-off of TCFM and TCDP, whereas the right panel shows how MAE_all and mean inference time change with different inference-step budgets. Across these settings, TCFM consistently achieves lower trajectory error at comparable or lower inference latency, indicating a more favorable accuracy-latency trade-off for target-conditioned short-horizon TCP approach generation.

The results show that the proposed flow-matching policy maintains a better accuracy-latency trade-off than the diffusion-policy baseline in the current task. Under a comparable inference-latency budget, TCFM with 10 integration steps requires 54.247 ms per inference and achieves an MAE_all of 0.0332, whereas TCDP with 10 denoising steps requires 67.292 ms and achieves an MAE_all of 0.0361. Moreover, as illustrated in **Figure 10**, increasing the diffusion sampling steps to 20 or 50 substantially increases inference cost without improving the final MAE in the current setting. These results suggest that, for target-conditioned short-horizon TCP approach generation, the proposed flow-matching formulation is more favorable in terms of accuracy-latency trade-off.

### 3.6 Failure cases and additional experimental findings

An additional phenomenon observed in the experiments is that a model trained mainly under low-light conditions shows reduced picking performance in strong-light background scenes. This observation is broadly consistent with findings in the fruit-harvesting robotics literature. Existing reviews indicate that illumination fluctuation, branch and leaf occlusion, fruit overlap, and the lack of local geometric information at the picking point can all strongly affect visual detection, picking-point localization, and visual-servo stability, and are among the main factors limiting robust deployment of fruit-harvesting robots (Kootstra et al., 2021; Huang et al., 2025). Similarly, autonomous harvesting systems for crops such as tomato have shown that, even when the overall system attains a relatively high success rate, the accuracy of perceiving stems, keypoints, and local structures in the perception front end still directly constrains downstream path planning and end-effector execution quality (Li et al., 2024). Therefore, the performance degradation observed here under strong illumination is more likely caused by the joint effect of reduced detection stability, distribution shift in visual features, and degradation of the policy condition representation, rather than by the failure of a single module.

**Figure 11:**
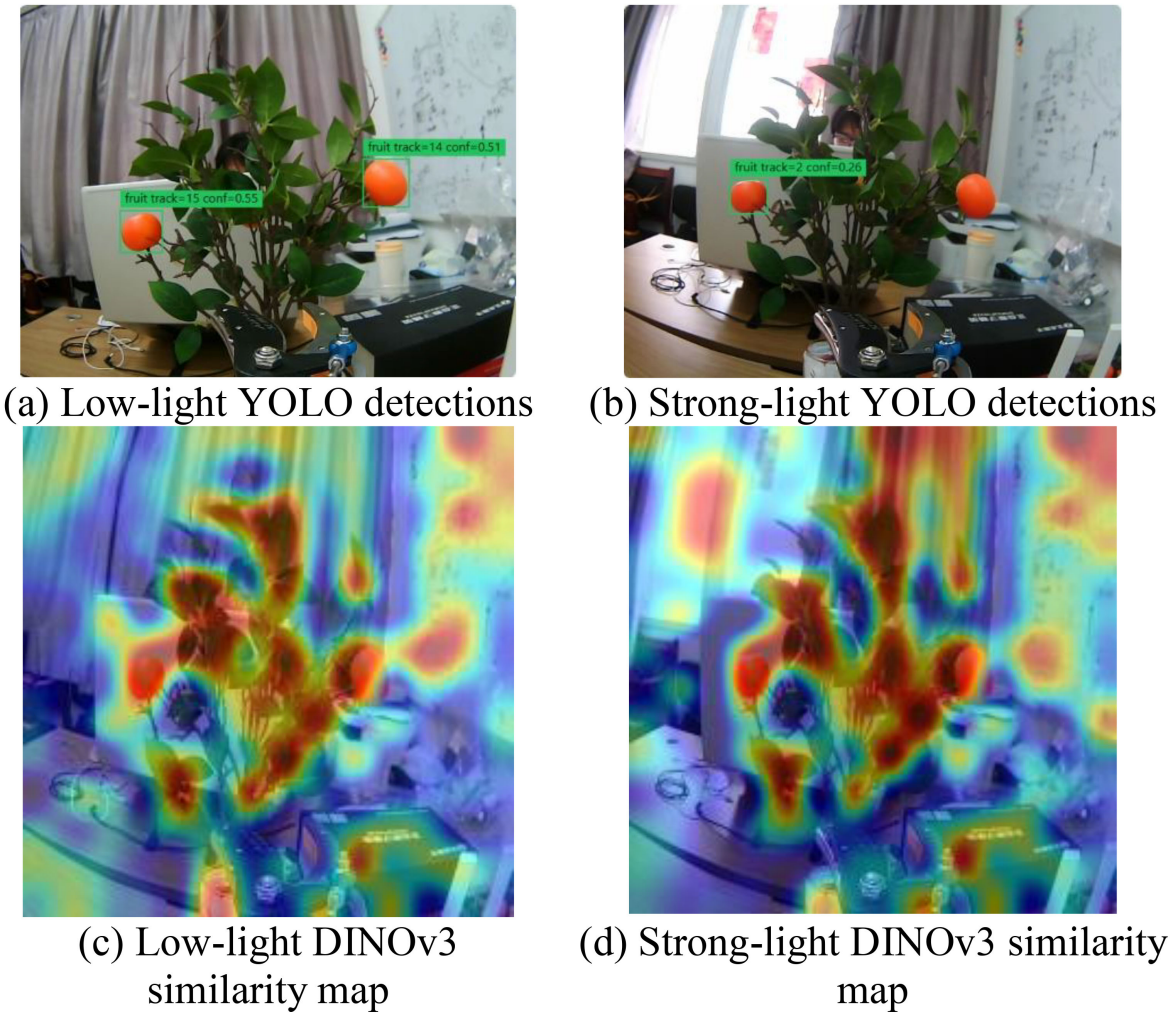
Comparison of similarity responses between YOLO detection results under different lighting backgrounds and DINOv3. **(a)** Low-light YOLO detections. **(b)** Strong-light YOLO detections. **(c)** Low-light DINOv3 similarity map. **(d)** Strong-light DINOv3 similarity map.

**Figures 11A, B** show that, after the illumination condition changes, both the confidence and stability of the YOLO detector decrease to some extent. At the same time, the DINOv3 similarity response maps in **Figures 11C, D** indicate that the visual backbone exhibits different response distributions in strong-light background regions under different lighting conditions. This illumination-induced perceptual shift is further propagated to the condition representation $c_{t}$ and the subsequent trajectory-generation process, thereby interfering with the Transformer’s attention allocation to key regions and ultimately affecting the execution quality of the predicted trajectory ${\hat{a}}_{t}$.

This phenomenon indicates that, although the proposed target-oriented augmentation alleviates appearance overfitting under small-sample conditions to a certain extent, the robustness of the model under clear cross-illumination shifts is still constrained by the performance of the perception front end. Additional experiments also show that supplementing the training set with strong-light background samples can partially alleviate the problem. This suggests that expanding the illumination coverage of the training data remains an effective way to improve robustness in deployment. Future work should therefore consider combining target-conditioned behavior learning with cross-domain detection, illumination normalization, or online visual adaptation mechanisms to improve stability in real agricultural environments.

### 3.7 Summary of experimental findings

The TCFM Policy can generate differentiated future trajectories ${\hat{a}}_{t}$ for different target conditions $g_{t}$ in the same scene, indicating that the explicit target-condition input can effectively reduce action ambiguity in multi-fruit scenes and enable target-conditioned path generation. In addition, the dual-branch visual representation and target-oriented augmentation improve policy performance from two complementary aspects: the ROI branch mainly improves final alignment accuracy, whereas target-oriented augmentation mainly reduces target loss and wrong-target picking. Their combination leads to higher overall online picking success. Finally, offline trajectory error can reflect how well the model fits the real future trajectory $a_{t}$ to some extent, but it is insufficient to fully characterize target consistency and task-completion quality. By contrast, the online success rate, target-picking error rate, and picking-point offset rate more directly reflect the practical value of target-conditioned behavior learning in real harvesting scenes.

The experimental results verify the effectiveness of the proposed method under small-sample, multi-target, and complex illumination conditions. They also show that combining explicit target conditions, dual-branch visual encoding, and conditional flow matching is a feasible strategy for improving visuomotor behavior learning in citrus harvesting robots.

## 4 Conclusion

This study proposes a target-conditioned flow-matching behavior-learning policy, namely TCFM Policy, for the pre-grasp approach stage of agricultural harvesting scenarios characterized by limited samples, multi-target interference, local occlusion, and fine end-effector manipulation requirements. The method is jointly designed from three aspects: target-constraint representation, visual representation construction, and multi-step trajectory generation. By introducing explicit geometric conditions of the target fruit, the policy can explicitly address the question of which fruit to pick. By combining global images and ROI images in a dual-branch visual representation and coupling this representation with target-oriented visual augmentation, the model improves both target perception and local operational robustness in cluttered foliage environments. In addition, the future TCP trajectory is modeled as a conditional flow-matching problem, enabling parallel generation of multi-step approach trajectories toward the specified target.

The real-world UR5 citrus harvesting demonstration dataset and the multi-fruit experiments show that the proposed method can generate differentiated approach paths for different targets within the same scene and can achieve comparatively high online picking success in the ablation study. The experiments further show that the ROI branch is important for improving picking-point arrival accuracy, whereas target-oriented augmentation is critical for reducing target loss and improving target consistency. At the same time, the offline trajectory analysis and failure cases indicate that average trajectory error alone does not fully represent harvesting performance, and perceptual shift under varying illumination remains an important factor affecting stable deployment.

The additional comparison with the TCDP baseline and the inference-step trade-off analysis further show that the proposed method achieves both lower trajectory error and a better accuracy-latency balance under matched training settings.

This work demonstrates the potential of combining target-conditioned behavior cloning with conditional flow matching for target-specified pre-grasp approach generation in agricultural harvesting robots and provides a new direction for end-to-end harvesting policy learning in real orchard environments. Future work will focus on cross-illumination and cross-scene generalization, joint optimization of target detection and policy learning, online closed-loop correction, and integrated grasping-and-cutting action modeling to improve continuous operation capability and engineering practicality in real orchards.

## Data Availability

The original contributions presented in the study are included in the article/supplementary material. Further inquiries can be directed to the corresponding author.
